# Sex-dependent effect of GPR109A gene deletion in myeloid cells on bone development in mice

**DOI:** 10.21203/rs.3.rs-6206075/v1

**Published:** 2025-04-02

**Authors:** Perry C. Caviness, Oxana P. Lazarenko, Michael L Blackburn, Jin-Ran Chen

## Abstract

Blueberry metabolite-derived phenolic acids are thought to suppress bone resorption via interactions with the G protein-coupled receptor 109A (GPR109A). Previously, global GPR109A knockout (GPR109A ^⁻/⁻^ ) mice exhibited increased bone mass and a diminished bone-protective response to phenolic acids. While GPR109A is highly expressed in osteoclast precursor macrophages, its role in bone development remains unclear. To address this, we generated a myeloid cell-specific GPR109A knockout (GPR109A ^flox/flox^ /LysM-Cre⁺; CKO) mouse model and assessed bone phenotypes in male and female mice at 35 days, 3 months, 6 months, and 12 months using µCT. At 35 days, CKO males showed significantly improved tibia and vertebrae µCT parameters compared to controls (f/f, Cre⁺). However, at later time points (6 and 12 months), Cre recombinase effects were observed, with Cre⁺ males exhibiting similar bone parameters to CKO mice. In contrast, female CKO mice displayed significantly improved µCT parameters at 6 and 12 months. Notably, 12-month-old Cre⁺ males exhibited altered bone mechanical properties, while females did not. Gene expression analysis revealed increased Interferon regulatory factor 8 (Irf8), an osteoclastogenesis suppressor, in female CKO mice. These findings suggest that GPR109A regulates bone resorption through osteoclastogenic pathways in a sex-specific manner.

## Introduction

GPR109A (HCAR2/HM74A/PUMA-G) is $G_i/G_o$ heterotrimeric G protein coupled receptor that has been reported to interact with a variety of biomolecules including short-chain fatty acids (SCFA) such as D-β-hydroxybutyric acid and β-hydroxybutyrate, butyric acid and butyrate [1]. In addition, GPR109A is also known to function as a receptor for niacin, mediating it's anti-lipolytic and anti-inflammatory effects [2, 3, 4, 5]. Upon binding with an agonist, GPR109A and other members of the $G_i/G_o$ family function inhibit adenylate cyclase, decreasing concentration of the secondary messenger cyclic adenosine monophosphate (cAMP) available, decreasing protein kinase A (PKA) activity leading to the expression or repression of a number of genes [6, 7]. Blueberry metabolite phenolic acids were significantly produced by gut microbiota in mice following 5% blueberry supplemented diet, specifically, hippuric acid (HA) and 3-(3-hydroxyphenyl) propionic acid (3-3-PPA) have been shown to interact with GPR109A, likely due to their structural similarities to niacin [3, 4, 8, 9]. HA and 3-3-PPA are known to promote increased bone formation and this effect is thought to be mediated at least partially through interactions with GPR109A [1, 10, 11, 12]. However, how GPR109A may play a role in regulating bone cell function and bone homeostasis is currently unknown.

Previously using a whole body GPR109A gene deletion mouse model, it was discovered that bone mass and strength were significantly increased in GPR109A$^{-/-}$ mice when compared to wild-type control mice at both 4-weeks and 6-months of age. In addition, bone resorption markers (TNFα, TRAP, Cathepsin K) were decreased and levels of bone formation marker P1NP were increased for GPR109A$^{-/-}$ mice compared to wild-type controls. In addition, in GPR109A$^{-/-}$ mice fed an HA supplemented diet, no significant changes were found in µCT parameters and bone resorption markers compared to

$GPR109A^{-/-}$ mice fed a control diet [1]. However, 5% blueberry diet was still shown to have an effect on μCT parameters and bone turnover markers in $GPR109A^{-/-}$ mice, suggesting additional factors from blueberry dietary supplementation than just HA and 3-3-PPA having bone protective qualities. $GPR109A^{-/-}$ was also found to ameliorate sex steroid deficiency induced bone loss in both male and female mice that had undergone ovari/orchiectomy. Bone resorption and formation markers were found to be significantly increased in sex steroid deficient wild-type mice but not in $GPR109A^{-/-}$ mice that had undergone ovari/orchiectomy [13].

GPR109A is expressed at high levels in osteoclast precursor macrophage cells, however the specific role GPR109A may play in bone cell homeostasis remains poorly understood [4, 14]. Using streptozotocin, mice with whole body gene deletion of GPR109A were found to be more susceptible to type I diabetes and had increased M1 macrophage polarization [15]. An additional study found that treatment of wild-type and whole body GPR109A gene deletion mouse models with nicotinic acid revealed significantly suppressed levels of inflammatory cytokines (TNF-α, IL-6, IL-12p40 and IL-1β) in wild-type bone marrow derived macrophages but did not affect inflammatory cytokine levels in macrophages isolated from $GPR109A^{-/-}$ mice suggesting GPR109A may serve as a negative regulator of macrophage activation. In the context of bone tissue, negative regulation of inflammatory cytokines may suppress osteoclastogenesis, further suggesting a role for GPR109A in regulating osteoclast differentiation [16, 17, 18, 19].

To investigate if GPR109A plays an important role in regulating bone turnover, via regulating differentiation of osteoclast precursor macrophages into mature osteoclasts, we have generated for the first time to our knowledge a myeloid cell (osteoclast precursor)-specific GPR109A CKO mouse model $GPR109A^{flox/flox}$/LysM-$Cre^{+}$ CKO ($GPR109A^{flox/flox}$/LysM-$Cre^{+}$). In this report, using μCT and three-point bending analysis we investigated change in bone phenotype in CKO mice compared to control mice ($GPR109A^{flox/flox}$, LysM-$Cre^{+}$) in both tibia and vertebrae from both sexes at different time points (35 days, 3 months, 6 months, 12 months). In addition, it was determined if myeloid cell-specific GPR109A gene deletion in mice affects gene and protein expression of osteoclastogenesis suppressor Interferon regulatory factor 8 (Irf8) in bone tissue. It is our hope that results from this study will enhance our understanding of GPR109A plays a role in regulating bone resorption and ultimately guide strategies for optimizing bone development.

### Results

## GPR109A myeloid cell specific deletion in mice

For myeloid cell specific deletion of GPR109A, targeting vector for incorporation of floxed GPR109A into mouse genome was used as previously described (Fig. 1A) [4, 20]. Male $GPR109A^{flox/+}$/LysM-$Cre^{+}$ and female $GPR109A^{flox/+}$/LysM-$Cre^{-}$ offspring were crossed to generate Myeloid cell specific GPR109A conditional knockout mouse models (CKO) as well as $GPR109A^{flox/flox}$/LysM-$Cre^{-}$ (f/f genotype) and

GPR109A$^{+/+}$/LysM-Cre$^{+}$ (Cre$^{+}$ genotype) (Fig. 1B). To confirm mouse genotypes tail DNA extraction and PCR was performed on mouse tails using primers designed to determine genotype (f/f, Cre$^{+}$, CKO) (Fig. 1C**)**. Primers used for genotyping are listed in Table 1. Agarose gel electrophoresis was performed to determine PCR product size (1.5% agarose gel, Ladder: DirectLoad Wide Range DNA Marker; D7058, Sigma).

Table 1
Primer sequences for mouse tail genotyping

| **Gene** | **Sequence (5'-3')** |
|---|---|
| GPR109A Forward | CTGCTTGGCTCGGAGCTTCTGTCTG |
| Gpr109A Reverse | AGGAAGAGGAGCTTCCTATGGAATG |
| LoxP site Reverse | CTGCTAAAGCGCATGCTCCAGACTG |
| LysM Forward | CTTGGGCTGCCAGAATTTCTC |
| LysM Reverse | TTACAGTCGGCCAGGCTGAC |
| LysM-Cre Reverse | CCCAGAAATGCCAGATTACG |

Immunohistochemistry was used to confirm deletion of GPR109A in bone tissue from 6-month-old mice. In f/f mice (both male and female) GPR109A protein (in red) is present in tibia alongside LysM (in green) with DAPI staining (in blue) used as control. For myeloid cell specific GPR109A CKO mice in tibia from both male and female mice, GPR109A protein levels are significantly depleted while LysM and DAPI staining appears unaffected compared to f/f mice. Best representative images of immunohistological results from f/f and myeloid cell specific GPR109A CKO tibia are presented in Fig. 2A and 2B.

### GPR109A/LysM-Cre genotype has sex dependent effect on tibia at different time points

To show the impact of myeloid cell specific deletion of GPR109A in long bone over time, µCT was performed to analyze trabecular bone in tibia isolated from male mice (f/f, Cre$^{+}$, CKO) at each timepoint (35 days, 3 months, 6 months, 12 months). At 35 days, myeloid cell deletion of GPR109A effect on bone appears limited as tibia images collected during µCT scans for f/f, Cre$^{+}$ and CKO mice appear identical to one another for male mice (**Supplemental Fig. 1A**). Of note, at 35-days male mice BMD for CKO genotype was significantly increased compared to both f/f and Cre$^{+}$ controls (35 days: male f/f N = 10, Cre$^{+}$ N = 10, CKO N = 10) (Fig. 3A). At 3 months, µCT scans for tibia show no change in trabecular bone for CKO male mice when compared to f/f and Cre$^{+}$ control mice (**Supplemental Fig. 1B**). At 3 months old no significant differences in µCT parameters for f/f, Cre$^{+}$ and CKO mice were detected with one exception. BMD for Cre$^{+}$ male mice were significantly increased compared to f/f but not CKO mice genotypes (3 months: male f/f N = 10, Cre$^{+}$ N = 10, CKO N = 9) (Fig. 3A). At 6 months, images collected from µCT scans for male tibia appear to show increased trabecular bone for CKO mice compared to f/f and $Cre^+$ control mice (**Supplemental Fig. 1C**). For 6-month-old males, Tb Sp, and BS/TV were improved for CKO mice compared to f/f controls but not $Cre^+$ mice. For male CKO mice, Tb Th was improved compared to both f/f and $Cre^+$ controls (6 month: male f/f N = 12, $Cre^+$ N = 12, CKO N = 12) (Fig. 3A). At 12 months, µCT collected images for male CKO mice show increased trabecular bone compared to f/f but not $Cre^+$ mice (**Supplemental Fig. 1D**). At 12 months BV, BV/TV, Tb N, Tb Sp and BS/TV were significantly improved for male CKO mice compared to f/f but not $Cre^+$ control mice (12 months: male f/f N = 11, $Cre^+$ N = 9, CKO N = 8) (Fig. 3A).

To best show the effects of CKO genotype in tibia trabecular bone over time, BMD was plotted for each group at each time point. For CKO mice at 35 days, BMD is significantly increased compared to f/f and $Cre^+$ mice but stays at or near this level for the remaining time points. BMD for f/f mice peaks at 6 months followed by a sharp decrease back to near 35-day levels for 12-month-old mice. For $Cre^+$ male mice, BMD peaks at 3 months and decreases back to near 35-day levels over the next 9 months (Fig. 3B).

As with male mice, µCT was performed on tibia isolated from female mice. At 35 days, µCT scanning show no significant change in trabecular bone between f/f, $Cre^+$ and CKO female mice (**Supplemental Fig. 1A**). In addition, no significant changes in µCT parameters were detected between genotypes (35 days: female f/f N = 9, $Cre^+$ N = 10, CKO N = 9) (Fig. 4A). At 3 months, µCT scans for female tibia show increased trabecular bone for CKO mice compared to f/f and $Cre^+$ control mice (**Supplemental Fig. 1B**). For female mice CKO mice, BV, Tb N and BS/TV were significantly improved compared to f/f but not $Cre^+$ control mice. BV/TV, Tb Sp and BMD were significantly improved for both $Cre^+$ and CKO mice compared to f/f control mice (3 months: female f/f N = 10, $Cre^+$ N = 5, CKO N = 10) (Fig. 4A). For female mice, at 6 months µCT collected images are similar to males, showing an increase in trabecular bone for CKO mice compared to f/f and $Cre^+$ control mice (**Supplemental Fig. 1C**). For 6-month-old female mice; BMD was significantly improved for CKO mice compared to both f/f and $Cre^+$ controls and Tb Sp as well as BS/TV were significantly improved for CKO mice compared to f/f but not $Cre^+$ controls (6 month: female f/f N = 12, $Cre^+$ N = 10, CKO N = 12) (Fig. 4A). For 12-month-old female mice, trabecular bone is increased for CKO mice compared to both f/f and $Cre^+$ controls (**Supplemental Fig. 1D**). For 12-month-old CKO female mice; BV, BV/TV, Tb N, Tb Sp, BS/TV and BMD were significantly improved compared to both f/f and $Cre^+$ control mice (12 months: female f/f N = 10, $Cre^+$ N = 7, CKO N = 9) (Fig. 4A).

As with male mice, BMD was plotted for each time point. At 35 days, BMD for female CKO mice is statistically similar to BMD for f/f and $Cre^+$ mice and reaches a maximum at 6 months before decreasing back to 35-day levels for 12-month-old mice. For $Cre^+$ female mice, BMD is significantly increased at 3 months, before being decreased to below 35-day levels at 12 months. For f/f female mice, BMD is statistically unchanged from 35 days to 6 months before being significantly decreased at 12 months compared to 6 months (Fig. 4B).

### GPR109A/LysM CKO has sex dependent effect on L3-L5 vertebrae at different time points

In addition to tibia, the effect of myeloid cell specific deletion of GPR109A on vertebrae was also investigated by performing μCT on L5 vertebrae isolate from f/f, $Cre^+$, and CKO mice from both sexes at each timepoint (35 days, 3 months, 6 months, 12 months). Based on images collected during μCT scanning; for 35-day-old male mice, L5 vertebrae trabecular bone appears to be increased in CKO mice compared to both f/f and $Cre^+$ control mice (**Supplemental Fig. 2A**). For 35-day old males; BV/TV, Tb N, Tb Sp and BS/TV were significantly improved for CKO mice compared to both f/f and $Cre^+$ controls. (35 days: male f/f N = 10, $Cre^+$ N = 10, CKO N = 10) (Fig. 5A). For 3-month-old male mice, there does not appear to be any changes in trabecular bone for CKO mice compared to either f/f or $Cre^+$ controls (**Supplemental Fig. 2B**). For 3-month-old male mice Tb N and BS/TV were significantly decreased for CKO mice compared to $Cre^+$ but not f/f controls (3 months: male f/f N = 10, $Cre^+$ N = 11, CKO N = 10) (Fig. 5A). At 6 months, μCT imaging of male L5 vertebrae appears to show increased trabecular bone for CKO and $Cre^+$ mice compared to f/f control mice (**Supplemental Fig. 2C).** For males, at 6 months Tb Th, BS/TV and BMD were significantly increased for CKO mice compared to f/f but not $Cre^+$ control mice. In addition, BS/TV for 6-month-old male $Cre^+$ mice was significantly increased compared to f/f control mice (6 months: male f/f N = 10, $Cre^+$ N = 10, CKO N = 10) (Fig. 5A). At 12 months, images collected during μCT of male L5 vertebrae appear to show increased trabecular bone for $Cre^+$ and CKO mice when compared to f/f controls (**Supplemental Fig. 2D**). For 12-month-old male mice, Tb Sp was significantly improved for CKO mice compared to f/f and $Cre^+$ control mice. In addition, Tb N and BS/TV were significantly increased for male CKO mice compared to f/f control, however for these parameters no significant difference between $Cre^+$ and CKO mice were detected (12 months: male f/f N = 11, $Cre^+$ N = 9) (Fig. 5A).

To better show the effects of GPR109A myeloid cell specific deletion in male vertebrae over time, BMD was plotted for each time point. For males, L5 vertebrae BMD is similar for f/f, $Cre^+$ and CKO mice at 35 days and is significantly increased for all three genotypes at 3 months. As with 35 days, BMD for f/f, $Cre^+$ and CKO mice is similar between the three genotypes at 3 months. However, from 3 months to 6 months BMD is increased for $Cre^+$ and CKO mice compared to f/f controls. From 6 months to 12 months, BMD for $Cre^+$ and CKO male mice is slightly decreased but greater than BMD for f/f mice (Fig. 5B).

As with male mice, μCT was also performed on L5 vertebrae isolated from female mice (f/f, $Cre^+$, CKO). For 35-day-old female mice, images collected during scanning of L5 vertebrae appear to show increased trabecular bone for CKO mice compared to both f/f and $Cre^+$ control mice (**Supplemental Fig. 2A**). For female mice at 35 days; BV/TV, Tb N and BMD were significantly improved for CKO mice compared to f/f but not $Cre^+$ mice. In addition, BMD for $Cre^+$ mice is also significantly increased compared to f/f control mice. Finally, at 35 days BS/TV is significantly decreased for CKO mice compared to $Cre^+$ control mice (35 days: female f/f N = 10, $Cre^+$ N = 10, CKO N = 10) (Fig. 6A). At 3 months, images from μCT of female

L5 vertebrae do not appear to show any changes in trabecular bone for CKO mice compared to either f/f or $Cre^+$ control mice (**Supplemental Fig. 2B**). For 3-month females, Tb N was significantly increased for both $Cre^+$ and CKO mice compared to f/f control mice. In addition, BV/TV and BS/TV were significantly increased for $Cre^+$ mice compared to f/f but not CKO mice. BV/TV and Tb N for CKO mice were significantly increased compared to both f/f and $Cre^+$ control mice. In addition, BV/TV and BMD are significantly increased for $Cre^+$ mice compared to f/f controls (3 months: female f/f N = 11, $Cre^+$ N = 10, CKO N = 10) (Fig. 6A). At 6 months, µCT imaging of female L5 vertebrae appears to show increased trabecular bone for CKO and $Cre^+$ mice when compared to f/f control mice (**Supplemental Fig. 2C**). For females, at 6 months BV/TV, Tb N, Tb Sp and BMD are significantly improved for CKO mice compared to both f/f and $Cre^+$ control mice. However, BV and BS/TV were significantly increased for both CKO and $Cre^+$ mice compared to f/f control mice (6 months: female f/f N = 10, $Cre^+$ N = 10, CKO N = 11) (Fig. 6A). For 12-month-old females, µCT collected images show increased trabecular bone for CKO mice compared to both f/f and $Cre^+$ control mice (**Supplemental Fig. 2D**). Finally, for female mice at 12 months; BV, BV/TV, Tb N and Tb Sp were significantly improved for CKO mice compared to both f/f and $Cre^+$ control mice. Interestingly, BS/TV was improved for CKO mice compared to $Cre^+$ mice but not f/f control mice (12 months: female f/f N = 10, $Cre^+$ N = 7, CKO N = 10) (Fig. 6A).

As with males, vertebrae BMD from female mice were plotted for each time point. For females, at 35 days BMD for $Cre^+$ and CKO mice is greater than BMD for f/f control mice. From 35 days to 3 months, BMD is increased for all genotypes however there were no significant differences in BMD between the genotypes at 3 months. From 3 months to 6 months, BMD for female CKO peaks and is significantly greater than BMD for f/f or $Cre^+$ mice. At 12 months BMD for f/f and $Cre^+$ mice is consistent with the levels at 3 and 6 months. While for CKO mice, BMD decreases back to 3-month levels (Fig. 6B).

### LysM-Cre male specific effect on femur biomechanical properties from 12-month-old mice

Three-point bending analysis of isolated femurs from f/f, $Cre^+$ and CKO mice; at 12 months, in both sexes; was performed to analyze the impact of myeloid cell specific GPR109A on bone biomechanical properties. For males, when compared to f/f control mice Yield Stress, Ultimate Stress and Elastic Modulus were significantly increased for both $Cre^+$ and CKO mice, with their being no significant difference in the presented three-point bending parameters between $Cre^+$ and CKO genotypes (Fig. 7A). However, for females, there is no significant difference detected for Yield Stress, Ultimate Stress or Elastic Modulus between ff, $Cre^+$ or CKO mice (Fig. 7B) (male: f/f N = 10, $Cre^+$ N = 8, CKO N = 8; female f/f N = 10, $Cre^+$ N = 7, CKO N = 10).

### Irf8 expression for 6-month-old CKO female mice is significantly increased

To investigate if GPR109A myeloid cell specific deletion will impact gene expression of osteoclast suppressive genes, Irf8 mRNA and protein levels were determined in L4 and L3 vertebrae isolated from 6-month-old male and female f/f, $Cre^+$ and CKO mice [21]. For males, Irf8 mRNA levels for f/f mice were increased compared to $Cre^+$ and CKO mice though not to a significant extent (Fig. 8A). Yet for females, Irf8 mRNA levels for CKO mice were significantly increased compared to both f/f and $Cre^+$ controls (Fig. 8B). For real-time PCR results N = 7 for both male and female samples across all genotypes. Western blots performed for protein isolated from L3-L5 vertebrae show a decrease in Irf8 protein levels for CKO male mice compared to both f/f and $Cre^+$ controls (Fig. 8C). While for female mice, western blots show significant increase in Irf8 protein levels for CKO mice compared to both f/f and $Cre^+$ controls (Fig. 8D).

## Discussion

Previously using a global GPR109A gene knockout mouse model, a significant increase in bone mass was observed for $GPR109A^{-/-}$ mice compared to wildtype controls. In addition, for $GPR109A^{-/-}$ mice protein and mRNA levels for osteoclastogenesis factors; including MMP9, NFATc1 and Cathepsin K were unchanged following 5% blueberry or HA dietary supplementation, suggesting a role for GPR109A in mediating the osteoclast suppressing effects of blueberry diet derived metabolites such as HA and 3-3-PPA [1, 10, 11, 12]. μCT results from this study seem to hint that myeloid cell specific GPR109A plays a role in regulating osteoclastogenesis, as deletion of GPR109A from these osteoclast precursor cells resulted in an improved bone phenotype (tibia and vertebrae) in both male and female mice depending on the timepoint measured [1]. It must be reiterated that these results only describe the bone phenotype and that further research will be required to understand the mechanisms by which myeloid cell specific GPR109A regulates osteoclastogenesis.

Myeloid cell specific deletion of GPR109A mouse model ($GPR109A^{flox/flox}$/LysM-$Cre^+$, CKO) was developed through breeding of female $GPR109A^{flox/flox}$ mice with male LysM-$Cre^+$. Offspring from this first breeding pair were then bred to each other to give the genotypes used for this experiment (f/f, $Cre^+$, CKO) [22]. Of particular note myeloid cell specific deletion of GPR109A revealed differences in bone phenotype between the different genotypes investigated for both sexes. For both tibia and L3-L5 vertebrae, at 6 and 12 months, trabecular bone and μCT parameters were in general improved for both male and female CKO mice compared to f/f control mice. Yet, male CKO mice μCT parameters were often found to be similar (not significantly different) to those from male $Cre^+$ mice unlike female CKO mice whose μCT parameters were significantly improved compared to both f/f and $Cre^+$ control mice at both 6 and 12 months. In addition to μCT parameters, three-point bending analysis results also show the same Cre recombinase specific male mice specific effect. Yield Stress, Ultimate Stress and Elastic Modulus in femur isolated from 12-month-old male mice showed significant improvements for $Cre^+$ and CKO genotypes when compared to f/f controls. However, unlike μCT results, 12-month-old female mice did not show significant changes in these three-point-bending parameters for f/f, $Cre^+$ or CKO mice. Both

µCT and three-point bending results for male mice hint at a sex specific effect for the $Cre^+$ genotype on bone.

Cre recombinase producing distinct phenotypes is a known phenomenon [23, 24, 25, 26]. In addition, a review of relevant literature shows that Cre recombinase male specific effects in mice have previously been observed. For instance, in Synapsin 1 (Syn1)-Cre rat models, used for investigating neuronal function, a significant increase in human growth hormone (HGH) transcription was observed in both male and female mice compared to wild-type controls. However only male Syn1-Cre mice exhibited decreased body weight and femur length likely through decreased liver Igf1 expression [27]. In addition, male Myosin heavy chain, α isoform (Myh6)-Cre mice, used for investigating cardiac function, were found to have decreased ejection fraction and left atrial dilation compared to control mice [28, 29]. While Cre male specific effects in mice have been observed, this is the first time to our knowledge that male LysM-$Cre^+$ mice and not female mice have had an effect bone phenotype comparable to CKO mice. The potential mechanism explaining LysM-$Cre^+$ genotype having increased bone mass in male, but not female mice may need to have further investigation.

It cannot be said for certain if the increase in trabecular bone and improved µCT parameters for myeloid cell specific GPR109A CKO ($GPR109A^{flox/flox}$/LysM-$Cre^+$) male mice at 6 or 12 months is due to deletion of GPR109A or the presence of Cre recombinase. However, at 35 days in male CKO mice tibia BMD and vertebrae BV/TV, Tb N and Tb Sp were significantly improved compared to both f/f and $Cre^+$ mice. These differences in µCT parameters were not nearly as apparent for female 35-day old CKO mice, suggesting a male specific effect for GPR109A on early life bone formation and development. Previous research has found that ketone bodies, including GPR109A substrate β-hydroxybutyrate, lower testosterone levels in young males [30]. G protein-coupled receptor family C group 6-member A (GPRC6A) has previously been shown to regulate testosterone production and energy metabolism [31]. It may be that GPR109A acts as a suppressor of testosterone production through unknown mechanisms. Yet it is unknown how myeloid cell deletion of GPR109A would impact testosterone production.

While the potential effects of GPR109A gene deletion in male mice appear to be primarily on early life bone development, in female mice, myeloid cell specific deletion of GPR109A effect on bone (tibia and L3-L5) is seen primarily at later time points (6 months, 12 months). Structural similarities between GPR109A and G-coupled protein estrogen receptor 1 (GPER1) as well as molecular docking studies, suggest that the two may share substrates including estrogens [32, 33, 34, 35]. GPR109A is normally associated with increased bone resorption, as such interactions between GPR109A and bone anabolic promoting substrates such as HA and 3-3-PPA are inhibitory [1, 10]. From this, we can hypothesis that in any potential interactions between estrogen and GPR109A reduce cAMP)/PKA signaling leading to increased osteoclastogenesis [10, 36, 37]. As such, myeloid cell deletion of GPR109A would promote increased bone formation through activation of cAMP/PKA signaling and increased downstream osteoclast suppressing effects of estrogen. GPR109A may also regulate expression of osteoclast suppressive gene *Irf8* in female mice, potentially explaining the sexual dimorphism observed in regard to bone density. However, the mechanism explaining the increase in *Irf8* expression for females CKO mice and male CKO mice is difficult to explain. The relationship between *Irf8* and GPR109A has not been fully explored in literature. Interestingly both, *Irf8* and *GPR109A* gene expression can be regulated by *Ifn-γ* and *Ifn-γ* gene expression can be regulated by estrogens [38, 39, 40]. However, if *Ifn-γ* is responsible for suppression of *Irf8* via GPR109A signaling, leading to weaker bones in females will need to be investigated further. Estrogen is thought to play a role in downregulating *Irf8* expression in osteoclast precursors through early estrogen induced gene 1 (EEIG1) [41]. As with *Ifn-γ* it is currently unknown if GPR109A signaling can regulate EEIG1 expression to explain sexual dimorphism observed in bone.

In this study the effect of myeloid cell specific (osteoclast precursor) deletion of GPR109A on bone phenotype was investigated at different time points (35 days, 3 months, 6 months and 12 months) in both male and females using for the first time to our knowledge a GPR109A/LysM CKO mouse model, hinting towards a role for GPR109A in regulating osteoclastogenesis. In early life GPR109A myeloid cell deletion was shown to significantly improve bone formation in male but not female mice. While at later ages, female GPR109 CKO mice had significantly improved μCT parameters. Significantly increased bone tissue expression of the osteoclast suppressive gene *Irf8* for 6-month old CKO female and not male mice suggest GPR109A signaling play a role in bone sexual dimorphism, however we can only speculate on mechanism how GPR109A and estrogen signaling may coordinate to suppress bone density and strength in females when compared to males. Future experiments will be performed to investigate how sex steroid signaling and GPR109A collaborate to explain sexual dimorphism present in bone. Further understanding of the osteoclastogenesis pathways regulated by GPR109A will assist in the development of novel treatments and therapeutics for alleviating high bone resorption disorders.

### Materials and Methods

## Production of GPR109A/LysM-CKO mouse model and animal care

Myeloid cell specific deletion of GPR109A in mice was accomplished using selective breeding. Briefly, female mice with loxP sites flanking GPR109A (GPR109A$^{flox/flox}$) were generated through a collaboration with Texas A&M Institute for Genomic Medicine and Emory University School of Medicine. GPR109A targeting vector was constructed by using PCR on full-length coding sequence of mouse GPR109A and cloning of amplified region (to be flanked by loxP regions) into pcDNA3.1 vector containing a gene for neomycin resistance flanked by Frt sites [4, 20]. Targeting vector was transformed into mouse embryotic stem cells via electroporation with loxP regions from targeting vector incorporated into genomic DNA via homologous recombination. Embryotic stem cells containing floxed GPR109A region are selected for using Neomycin and injected into C57BL/6 embryos. Backcrossing of chimeric GPR109A$^{flox/flox}$ mice with C57BL/6 mice was performed to develop stable GPR109A$^{flox/flox}$ mouse line [4]. Deletion of GPR109A in myeloid cell line (monocytes, mature macrophages, osteoclasts) was accomplished through breeding of female GPR109A$^{flox/flox}$ mice with male mice containing the coding sequence for Cre recombinase inserted into the first ATG codon of the lysozyme 2 gene (LysM), an antibacterial enzyme expressed in myeloid cells (LysM-Cre$^{+}$), Jackson laboratory, B6.129P2-Lyz2$^{tm1(cre)Ifo}$/J, Strain #:004781). From the offspring male GPR109A$^{flox/+}$/LysM-Cre$^{+}$ and female GPR109A$^{flox/+}$/LysM-Cre$^{-}$ mice were interbred based on previously published methods to generate the following genotypes; wild-type, GPR109A$^{flox/+}$/LysM-Cre$^{-}$, GPR109A$^{flox/flox}$/LysM-Cre$^{-}$ (f/f), GPR109A$^{+/+}$/LysM-Cre$^{+}$ (Cre$^{+}$), GPR109A$^{flox/+}$/LysM-Cre$^{+}$ and GPR109A$^{flox/flox}$/LysM-Cre$^{+}$ (CKO) [22]. For this study only Cre$^{+}$, f/f and CKO mice were used and additional breeding was performed as necessary to generate these needed genotypes.

Following generation of needed genotypes. Mice were weighed and randomized into 4 different lifespan groups (35 days, 3 months, 6 months, 12 months). Mice were housed 6 per cage in small shoe box cages and fed a purified control diet. Mice were weighed weekly and weight/mouse at the completion of lifespan are listed in supplemental file 1. After the completion of lifespans, mice were euthanized via inhalation of $CO_2$ followed by exsanguination. Tibia and L3-L5 vertebrae from mice were collected and stored at -80 ºC in formalin. Mice were housed in an Association for Assessment and Accreditation of Laboratory Animal Care-approved animal facility in the Arkansas Children’s Nutrition Center Animal Studies Core at the Arkansas Children’s Research Institute, with constant humidity and lights on from 06:00–18:00 hrs. at 22°C. All animal procedures were approved by the Institutional Animal Care and Use Committee at University of Arkansas for Medical Sciences (AUP#3595 UAMS, Little Rock, AR).

## Mouse tail genotyping

Genotyping of offspring mice was performed on mice tails using Extract-N-Amp PCR kit (Sigma-Aldrich). Briefly, newborn mice were anesthetized using isoflurane and end of tails were snipped and collected. 50 µl of extraction solution and 12.5 µl of preparation solution were added to microcentrifuge tubes containing tails. Samples were first incubated at 55° C for 1 hr. followed by incubation 95° C for 5 min. Following incubation, 50 µl of neutralization solution was added and PCR was performed for both Cre recombinase and GPR109A$^{flox/flox}$. Primers for LysM-Cre and floxed GPR109A CDS are listed in Table 1. Genotypes of offspring mice were determined based on PCR product size using Jackson Laboratory protocols.

## Bone immunohistology analysis

Mouse right tibia samples were decalcified using EDTA, embedded, cut and slides were prepared by Histology Special Procedures at the Arkansas Children's Nutrition Center Histology Core. Slides for f/f and CKO mouse genotypes were washed with 1X PBS (10–15 min., room temperature). PBS was removed and 2.5% Horse Blocking serum was added (20 min., room temperature). Blocking serum was removed via aspiration and bone tissue slides were incubated with primary antibody (GPR109A: A02511, Boster LysM: 66456-1-Ig; Proteintech) diluted 1:50 in 2.5% horse blocking serum containing 1% IGEPAL overnight at 4° C. Slides were washed with 1X PBS containing 0.05% IGEPAL (3 min., 3 times at room temperature) and secondary antibody was added (Goat anti-Rabbit IgG Secondary Antibody, Alexa Fluor 647; Goat anti-mouse IgG Secondary antibody, AlexaFluor 546) (1 hr., room temperature, protected from light). Final slides were covered with DAPI-Fluoromount-G and observed using Nikon Eclipse T/2 epifluorescent microscope.

## µCT scan of GPR109A/LysM-cKO tibia and L3-L5 vertebrae

Micro-computed tomography (CT) measurements of tibia and L5 vertebrae from CKO, $Cre^+$ and f/f mice (all age groups) were evaluated using a Skyscan µCT scanner (SkyScan 1272.). Tibia and vertebrae were cleaned of muscle tissue and stored in formalin for at least 24 hrs. prior to scanning. For µCT scanning and analysis of both tibia and vertebrae trabecular bone, the region of interest (ROI) was selected to include the region extending 0.9 mm distally and 0.03 mm from the physis [42]. Images were obtained at 70 kV X-ray tube voltage and 142 µA current, from a 0.5 mm aluminum filter. Images were reconstructed using NRecon software (Skyscan). Random movement and flat field correction were turned on and beam hardening correction was set to 38%. Total bone volume (BV $mm^3$), tissue volume (TV $mm^3$), bone volume fraction (BV/TV %), bone surface (BS/TV mm2), trabecular thickness (Tb Th, mm), trabecular separation (Tb Sp, mm), trabecular number (Tb N, 1/mm) and bone mineral density (BMD $g/cm^3$) were calculated using Skyscan provided software and averaged for each age group

## Three-point bending of GPR109A/LysM-cKO femur

Before mechanical testing, right femurs were thawed and re-wrapped in wet gauze then scanned using micro computed tomography (µCT: Bruker Skyscan 1272, Billerica, MA). Images were obtained at 60 kV X-ray tube voltage and 166 µA current, using a 0.5 mm aluminum filter, 1026 ms exposure time, and 25.90 µm image pixel size. For each specimen, a series of 628 projection images were obtained (a rotation step 2.0°, averaging 3 frames). Images were reconstructed to obtain images using NRecon software (Skyscan). Next, images were subjected to morphometric analysis using CTAn software (CT Analyser 1.13.5.1, Skyscan). Average minimum moment of inertia and centroid were calculated from 100 slices centered at the mid-diaphysis. Following µCT scanning, right femurs were loaded to failure in three-point bending using a Z2.5 material testing machine with an XForceP 0.2 KN load cell (Zwick/Roell, Ulm, Germany). The fixed distance between the lower supporting bars was 6.82 mm, with a displacement rate of 0.5 mm/min. The anterior mid-diaphysis was pre-loaded at 0.5 N, and the tests were analyzed using the load displacement curve created by the system's analysis software TestXpert III (Zwick/Roell, Version 1.6). Yield was defined as the point at which the regression line that represents a 5% loss in stiffness crosses the load displacement curve.

## RNA isolation, real-time reverse transcription-polymerase chain reaction

RNA was isolated from L3-L5 vertebrae of 6-month-old mice (f/f, $Cre^+$, CKO; male and female) by homogenization in trizol with metal beads and Precellys 24 homogenizer (6500 rpm, 20 seconds, twice). After homogenization total RNA was purified using RNeasy plus Mini Kit. RNA concentration and purity (A260/A280) for RNA samples was determined using a Polarstar Omega plate reader. Reverse transcription was carried out using an iScript cDNA Synthesis Kit from Bio-Rad. Real-time polymerase chain reaction (RT-PCR) experiments for analyzing mRNA levels of *Irf8* were carried out using SYBR Green and with the QuantStudio 6 Flex real-time PCR system from Applied Biosystems. For each sample, RT-PCR was performed in duplicate and averaged. Gene expression data was normalized to *Cyclophilin A*

## SDS PAGE & Western blot

SDS-PAGE and western blot was performed to determine change in Irf8 protein levels in 6-month-old mice L3-L5 vertebrae (f/f, $Cre^{+}$, CKO; male and female). Total protein was isolated by homogenization with RIPA buffer (Solarbio) using metal beads Precellys 24 homogenizer (6000 rpm, 40 seconds). Following homogenization, samples were incubated on ice for 45 min (samples vortexed every 10 min). Following incubation of ice, samples were centrifuged (14000 x G, 15 min) and supernatant was collected as bone tissue protein lysate. Protein concentrations were found for lysates using BCA assay. Samples were prepared for SDS PAGE (6X SDS-sample buffer, Boston BioProducts, BP-111NR) on 10% Acrylamide-Bisacrylamide gel. Irf8 recombinant mouse primary antibody was used (Invitrogen, 39-8800) with HRP Conjugated goat anti-mouse IgG (R&D systems, HAF018) as the secondary antibody. β-actin mouse primary antibody (A1978, Sigma-Aldrich) was used for loading control. Bands of interest were visualized and imaged under chemiluminescent detection using the iBright 1500 system from ThermoFisher Scientific. For western blot, 2 protein lysate samples from each genotype were randomly combined to give 1 pooled sample. 3 pooled samples were used for each genotype.

## Statistical Analysis

For experiments, numerical variables were expressed as mean +/- SD (Standard Deviation); n equals to the number of samples/group. Statistical analysis was performed with GraphPad Prism 9.0 (GraphPad Software, Inc., San Diego, Ca, USA). Outliers were determined using ROUT method (Q = 10%) and removed [43]. Differences within groups were evaluated using one-way ANOVA and corrected for multiple comparisons by Tukey post hoc test. We confirm that the study is reported in accordance with ARRIVE guidelines.

### Declarations

## Disclosures

The authors declare no conflicts of interest.

## Funding:

This study is supported by USDA-ARS Project 6026-10700-001-000D; and NIH R01 project R37 AA18282 sub-awarded to JRC.

We confirm that all methods/animal experiments were carried out in accordance with relevant guidelines and regulations.

## Author Contribution

J.R.C. designed the study; P.C.C. wrote the original draft and produced figures; J.R.C., and P.C.C. revised and edited the paper; O.P.L. and M.L.B. performed ex vivo experiments; P.C.C., J.R.C., O.P.L. and M.L.B. analyzed results. All authors reviewed the manuscript.

## Acknowledgements

Authors would like to thank Jim Sikes, Hoy Pittman and Bobby Fay for their technical assistances on animal experiments. This work was supported by sub-objective to J.R.C. by United States Department of Agriculture (USDA) / Agricultural Research Service (ARS) Project # USDA-ARS Project 6026-51000-012-06S as well National institute of health project R37 AA18282 sub-awarded to J.R.C. Authors declare that they have no competing interests. All data are available in the main text or the supplemental materials.

## Data Availability

The data that support the findings of this study are available in the methods and/or supplemental material of this article.

## Figures

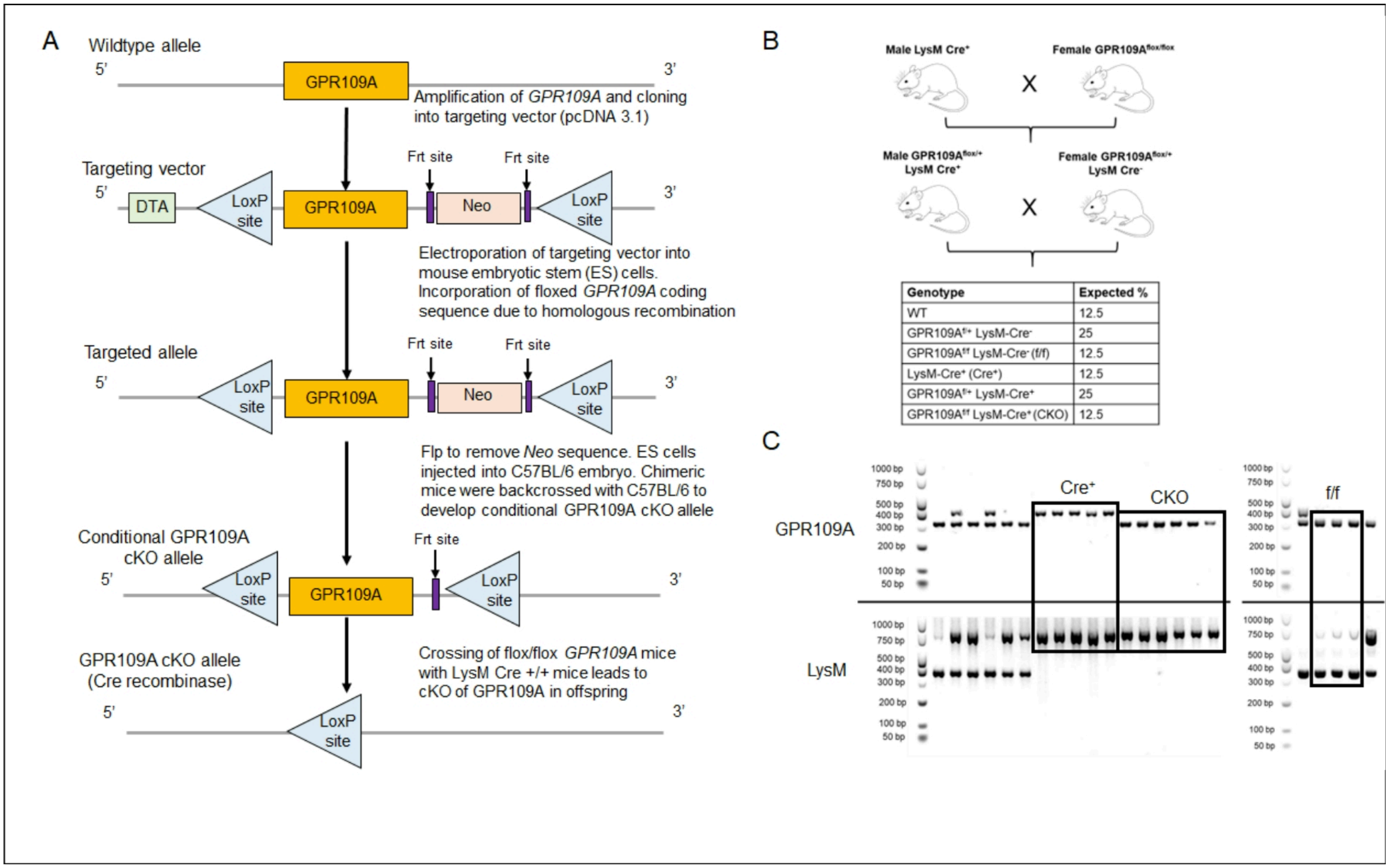

### Figure 1

Targeting vector and breeding strategy for generation of myeloid cell specific GPR109A CKO ($GPR109A^{flox/flox}$/LysM-$Cre^{+}$) mouse model. **A:** GPR109A CDS was cloned into pcDNA3.1 vector whose cloning site is flanked by LoxP regions. Floxed GPR109A region was incorporated into mouse embryotic stem cell allele via electroporation and homologous recombination. Embryotic stem cells carrying floxed

GPR109A CDS were injected into C57BL/6 embryos which were carried to term. Adult mice carrying floxed GPR109A CDS were backcrossed to develop a stable mouse line carrying a conditional GPR109A CKO allele. **B:** Female GPR109A$^{flox/flox}$ mice were crossed with male LysM-Cre$^{+}$ mice to give offspring with either GPR109A$^{flox/+}$/LysM-Cre$^{+}$ or GPR109A$^{flox/+}$/LysM-Cre$^{-}$ genotypes. Male GPR109A$^{flox/+}$/LysM-Cre$^{+}$ and female GPR109A$^{flox/+}$/LysM-Cre$^{-}$ offspring were crossed to give Cre$^{+}$, f/f and CKO genotypes as well as others needed for this study. **C:** Genotyping was performed on mouse tails using PCR followed by agarose gel electrophoresis (1.5%). Primers were designed to detect the presence (350 bp) or absence (400 bp) of floxed regions flanking GPR109A CDS as well as presence of LysM-Cre (750 bp) or Cre (350 bp) in DNA isolated from mouse tail samples.

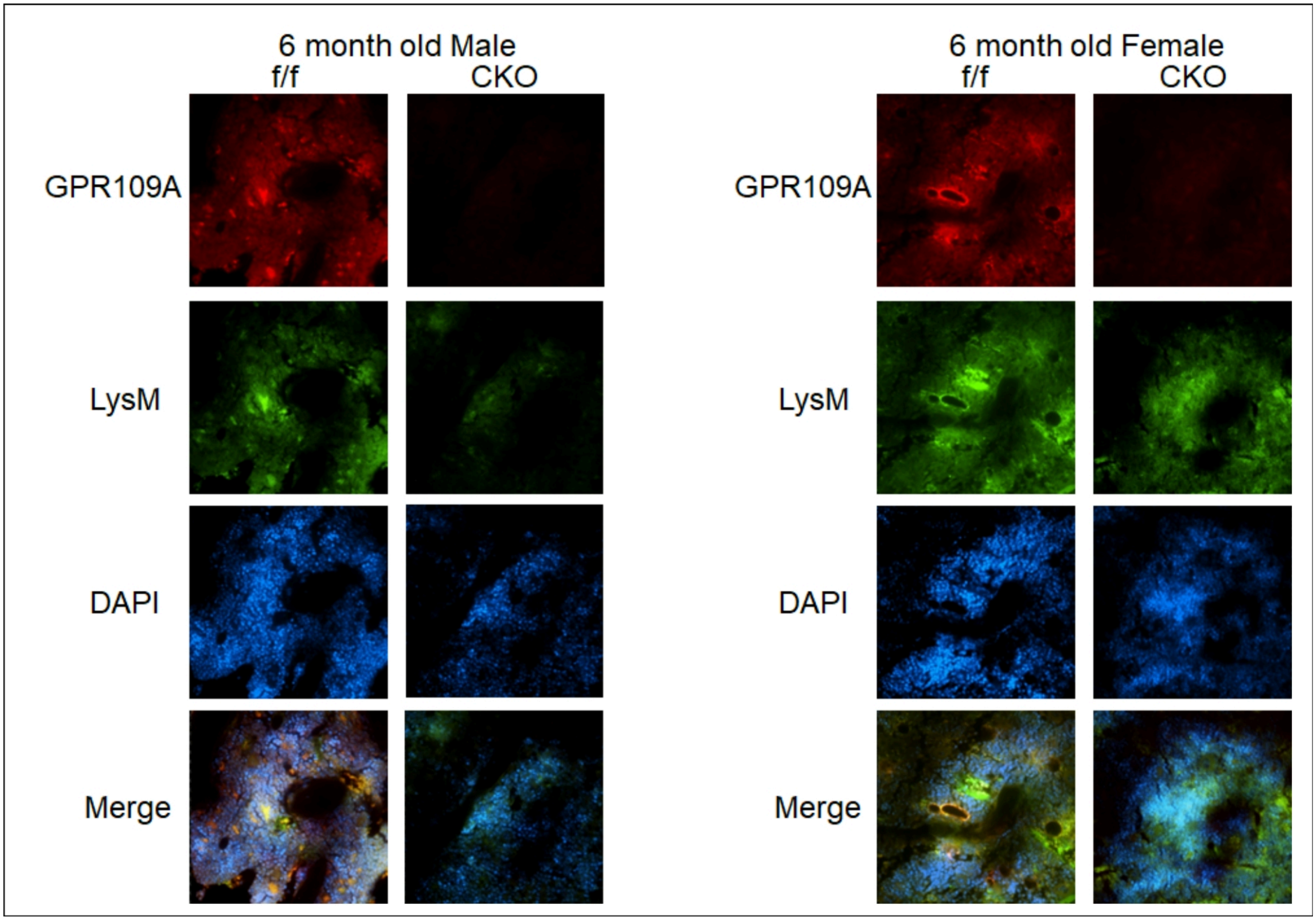

### Figure 2

Best representative images from immunohistochemistry confirm deletion of GPR109A in tibia of both male and female CKO mice. **A & B:** Immunohistochemistry was performed on tibia isolated from male and female f/f and CKO mice at 6 months of age. Loss of GPR109A protein expression (in red) was confirmed for CKO mice is present in tibia as well as the presence of LysM-Cre (in green). LysM and DAPI staining (in blue) are unaffected for CKO mice compared to f/f mice confirming myeloid cell deletion of GPR109A did not affect tibia at the cell level.

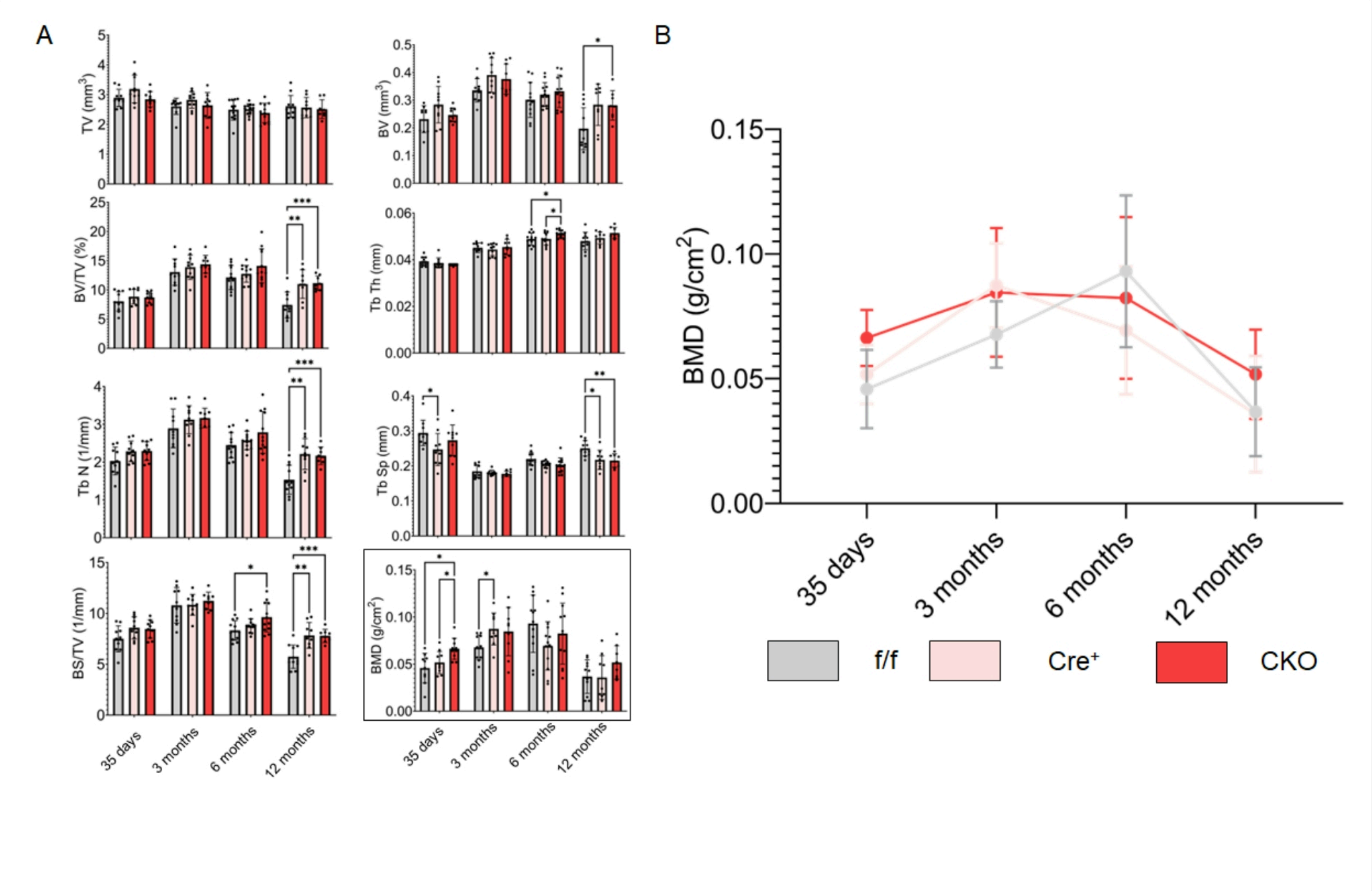

### Figure 3

μCT analysis of male tibia reveals early life improvements in trabecular bone for CKO mice compared to f/f and $Cre^+$ controls. **A:** μCT parameters for male tibia (f/f, $Cre^+$ and CKO) at different time points (35 days, 3 months, 6 months, 12 months). BMD was significantly increased at 35 days for CKO mice compared to f/f and $Cre^+$ controls. Yet at 12 months, Cre recombinase effect is observed as BV/TV, Tb N, Tb Sp and BS/TV are significantly improved for both CKO and $Cre^+$ mice compared to f/f mice. **B:** Line graph of tibia BMD over all time points shows that early life BMD increase for CKO mice compared to other genotypes is not sustained throughout life. For One-way ANOVA, *P ≤ 0.05, **P ≤ 0.01, ***P ≤ 0.001, **** P ≤ 0.0001.

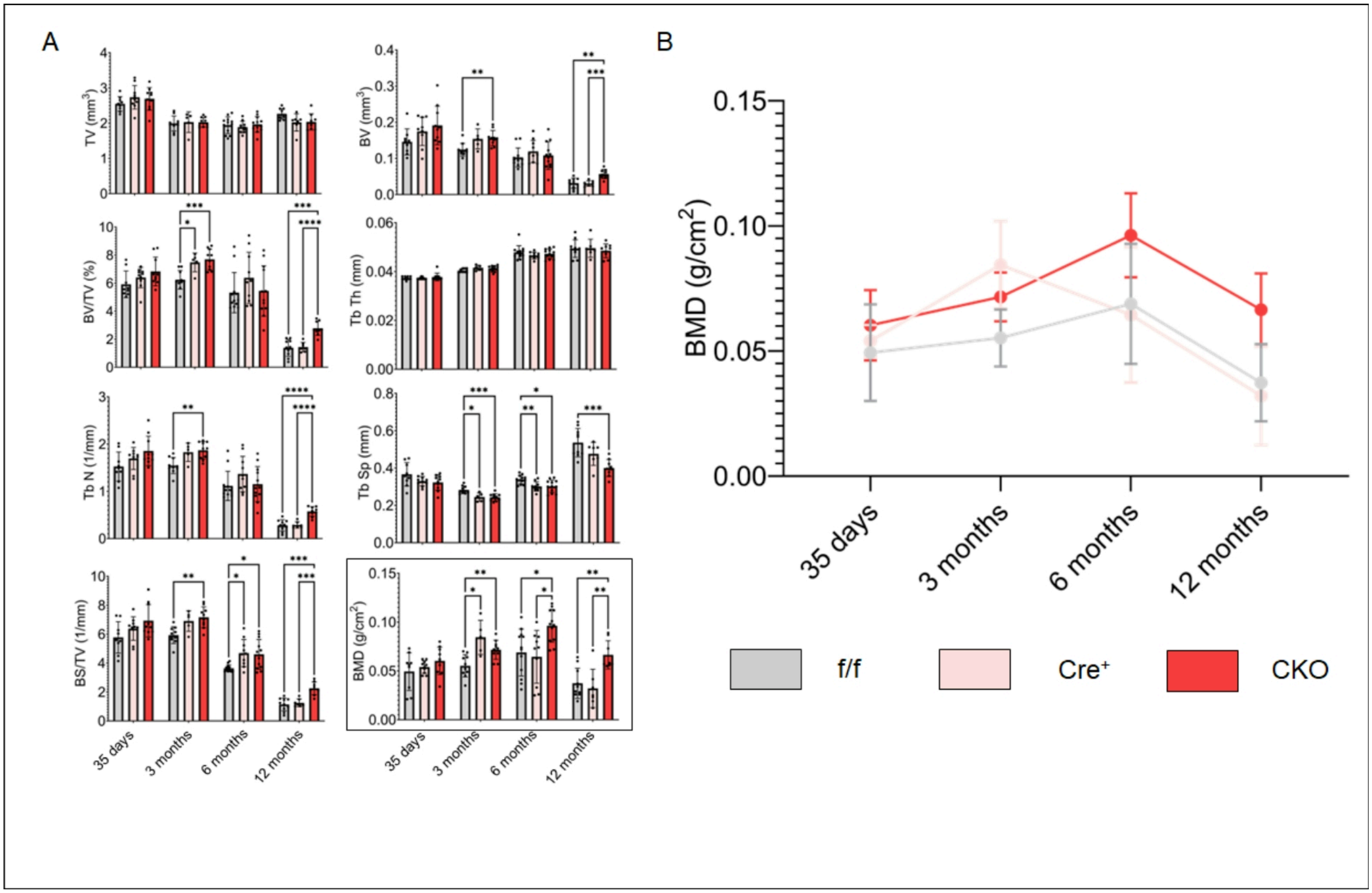

**Figure 4**

μCT analysis of female tibia reveals later in life improvements in trabecular bone for CKO mice compared to f/f and $Cre^{+}$ controls. **A:** μCT parameters for female tibia (f/f, Cre+ and CKO) at different time points (35 days, 3 months, 6 months, 12 months). For female mice, impact of GPR109A myeloid cell deletion is primarily observed at 12 months as evident by significant increases in BV, BV/TV, Tb N, BS/TV and BMD for CKO mice compared to both f/f and $Cre^{+}$ mice. For CKO mice, BMD is also significantly improved at 6 months when compared to both f/f and $Cre^{+}$ controls. **B:** Line graph of female mice tibia BMD over all time points showing peak BMD occurs at 6 months for CKO mice. For One-way ANOVA, *P ≤ 0.05, **P ≤ 0.01, ***P ≤ 0.001, **** P ≤ 0.0001.

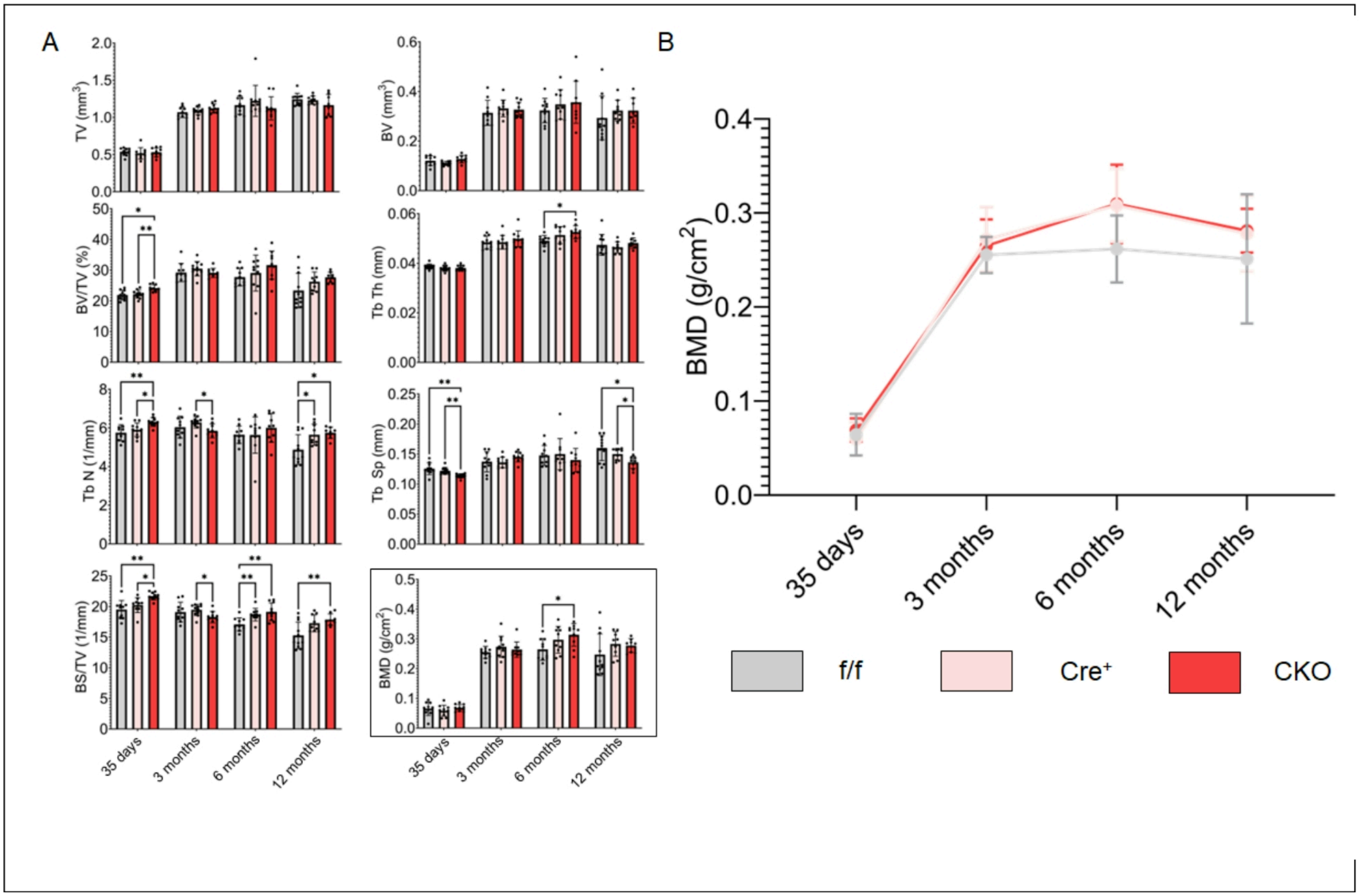

### Figure 5

µCT analysis of male L5 vertebrae reveal early life improvements in CKO trabecular bone compared to f/f and Cre$^+$ controls. **A:** µCT parameters for male L5 vertebrae (f/f, Cre$^+$ and CKO) at different time points (35 days, 3 months, 6 months, 12 months). BV/TV, Tb N, Tb Sp and BS/TV are significantly improved for CKO mice at 35 days compared to f/f and Cre$^+$ controls. Cre recombinase effect was also observed at latter time points as BS/TV is significantly increased for both Cre$^+$ and CKO 6-month mice compared to f/f and Tb N is significantly improved for Cre$^+$ and CKO mice compared to f/f at 12 months. In addition, at both 6 and 12 months, BMD for Cre$^+$ and CKO are similar, and both are increased compared to f/f mice but not to a significant extent. **B:** Line graph of male L5 vertebrae BMD over all time points shows no change between genotypes at early time points but similar increases for both Cre$^+$ and CKO mice compared to f/f at 6 and 12 months. For One-way ANOVA, $*P \leq 0.05$, $**P \leq 0.01$, $***P \leq 0.001$, $****P \leq 0.0001$.

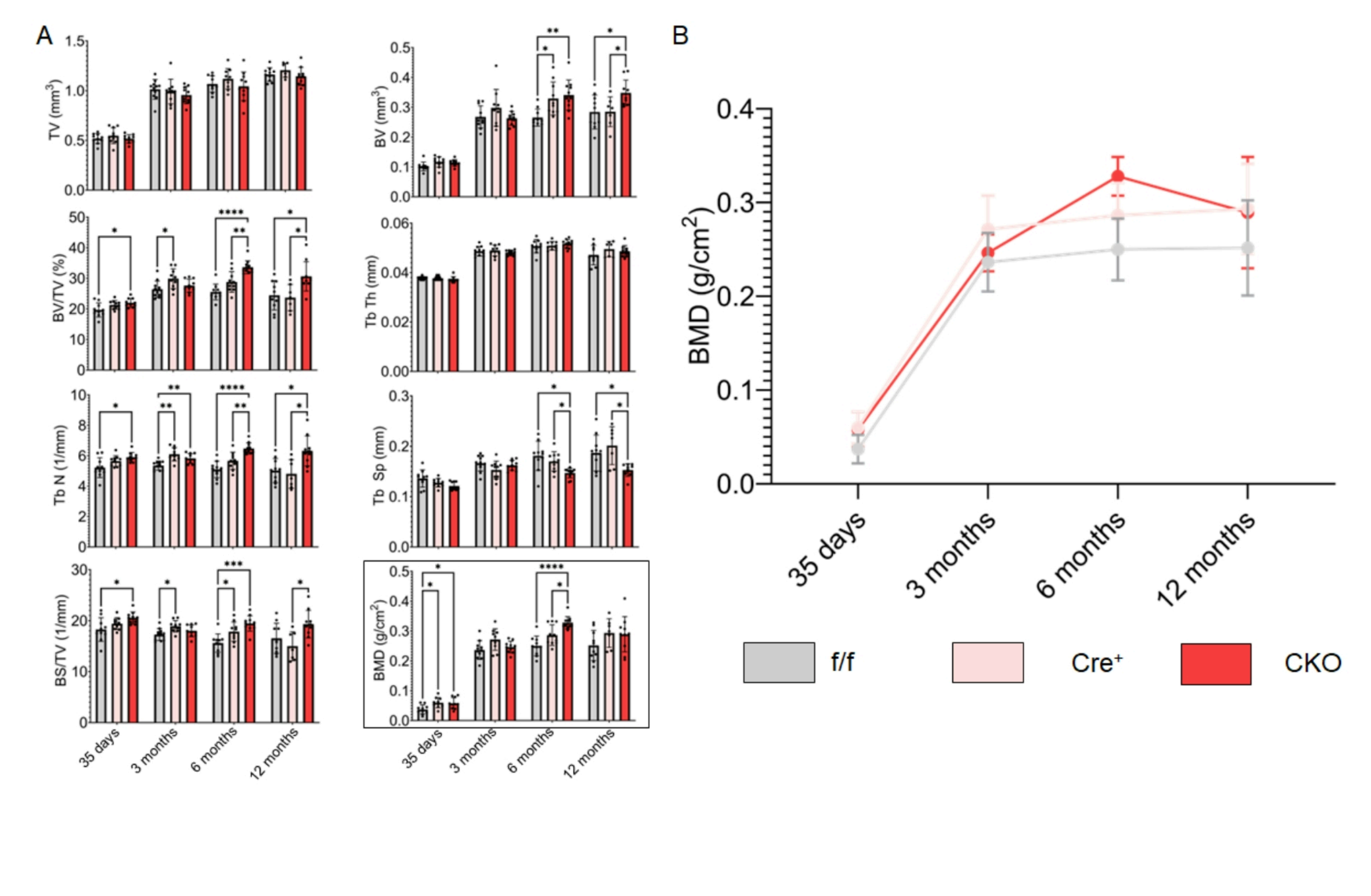

### Figure 6

µCT analysis of female L5 vertebrae reveal later in life improvements in CKO trabecular bone compared to both f/f and Cre$^+$ controls. **A:** µCT parameters for L5 vertebrae from female mice (f/f, Cre$^+$ and CKO) at different time points (35 days, 3 months, 6 months, 12 months). Impact of GPR109A myeloid cell deletion is primarily observed at later time points as observed by significant improvements in BV/TV, Tb N and Tb Sp at both 6 and 12 months for CKO mice compared to both f/f and Cre$^+$; while BMD for CKO mice is significantly improved compared to f/f and Cre$^+$ controls at 6 months. **B:** Line graph of female L5 vertebrae BMD over all time points showing peak BMD occurs at 6 months for CKO mice. For One-way ANOVA, *P ≤ 0.05, **P ≤ 0.01, ***P ≤ 0.001, **** P ≤ 0.0001.

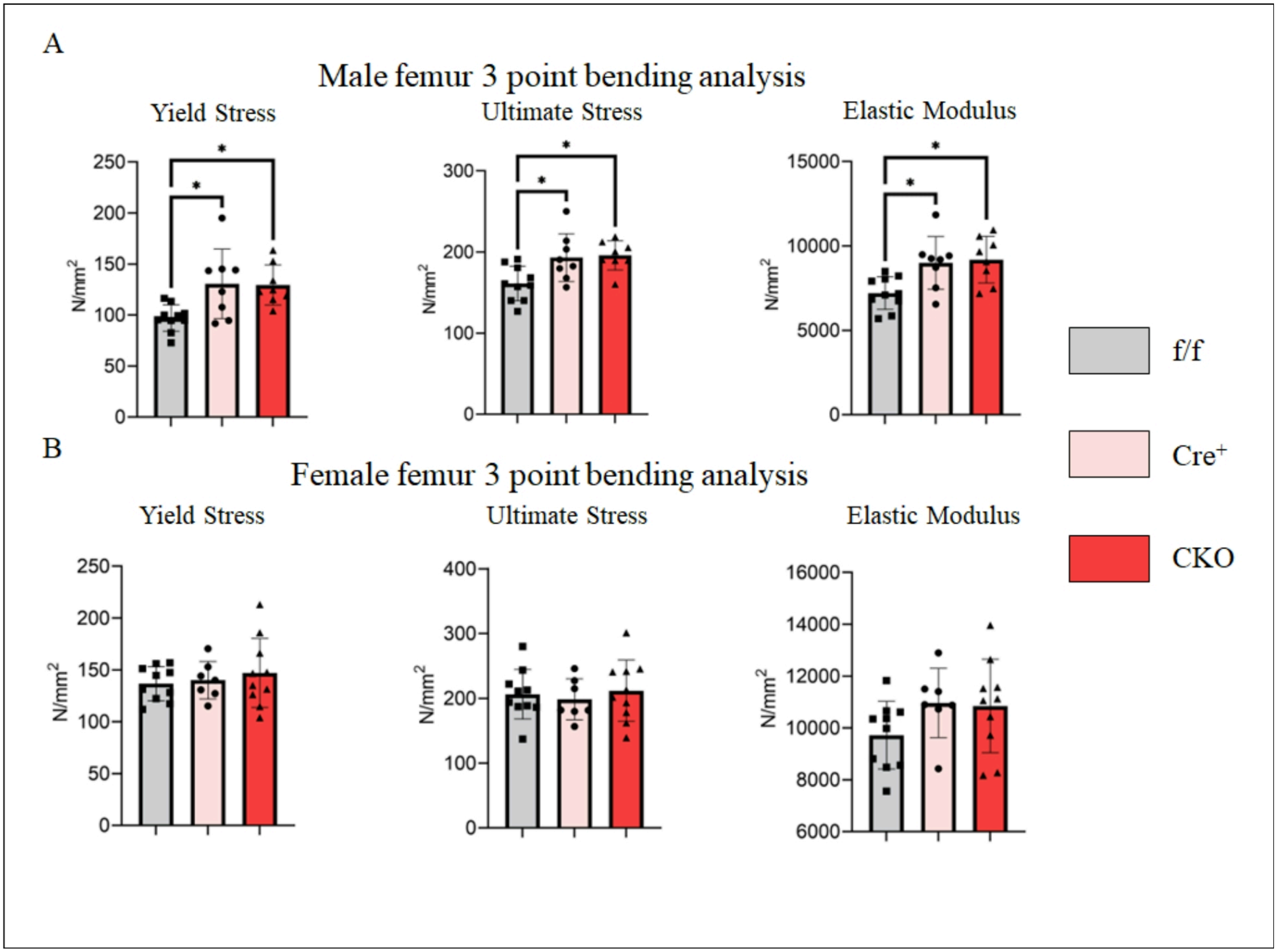

### Figure 7

Cre recombinase male specific effect is also observed in 3-point bending analysis results. **A:** For males, 3-point bending analysis of femur from 12-month old mice reveals significant increases in Yield Stress, Ultimate Stress and Elastic Modulus for both $Cre^{+}$ and CKO mice compared to f/f mice. **B:** For females, 3-point bending analysis of 12-month old femur shows no significant changes in Yield Stress, Ultimate Stress and Elastic Modulus between f/f, $Cre^{+}$ or CKO mice. For One-way ANOVA, *P ≤ 0.05, **P ≤ 0.01, ***P ≤ 0.001, **** P ≤ 0.0001.

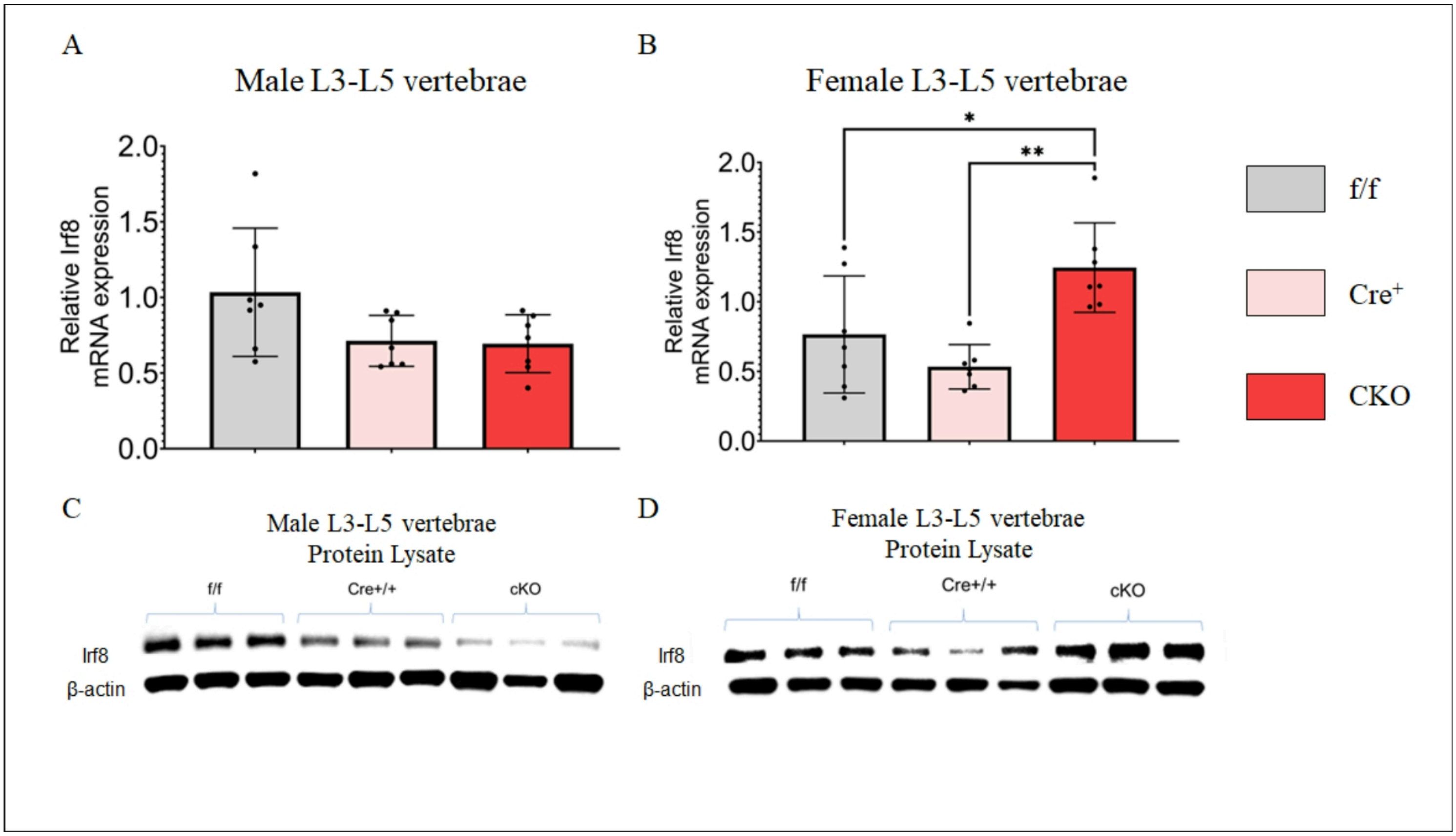

**Figure 8**

Irf8 mRNA and protein levels in bone tissue from 6-month old vertebrae are significantly increased for only female myeloid specific GPR109A CKO mice. **A:** Real-time PCR results for RNA isolated from 6-month old male L4 vertebrae show no significant changes in Irf8 mRNA levels between f/f, $Cre^+$ and CKO mice. **B:** Real-time PCR results for RNA isolated from 6-month old female L4 vertebrae show a significant increase in Irf8 mRNA for CKO mice compared to f/f and $Cre^+$ controls. **C:** Western blot of protein lysate from male L3 vertebrae shows a decrease in Irf8 protein for CKO mice compared to both f/f and $Cre^+$ controls. **D:** Western blot of protein lysate from female L3 vertebrae shows an increase in Irf8 protein for CKO mice compared to both f/f and $Cre^+$ controls. For western blot, 2 protein lysate samples from each genotype were randomly combined to give 1 pooled sample. 3 pooled samples were used for each genotype.

## Supplementary Files

This is a list of supplementary files associated with this preprint. Click to download.

- SupplementalFigure122025manuscriptfigures.pptx
- CopyofSupplementalfile1.xlsx
- SupplementalFigure222025manuscriptfigures.pptx
